# Intracytoplasmic Sperm Injection May Not Improve Clinical Outcomes Despite Its Positive Effect on Embryo Results: A Retrospective Analysis of 1130 Half-ICSI Treatments

**DOI:** 10.3389/fendo.2022.877471

**Published:** 2022-06-15

**Authors:** Nan Peng, Shuiying Ma, Cheng Li, Hui Liu, Haibin Zhao, Lian-Jie Li, Qing Li, Mei Li

**Affiliations:** ^1^ Center for Reproductive Medicine, Cheeloo College of Medicine, Shandong University, Jinan, China; ^2^ Key Laboratory of Reproductive Endocrinology of Ministry of Education, Shandong University, Jinan, China; ^3^ Shandong Key Laboratory of Reproductive Medicine, Jinan, China; ^4^ Shandong Provincial Clinical Research Center for Reproductive Health, Jinan, China; ^5^ National Research Center for Assisted Reproductive Technology and Reproductive Genetics, Shandong University, Jinan, China; ^6^ The Second Hospital, Cheeloo College of Medicine, Shandong University, Jinan, China

**Keywords:** half-ICSI, 2PN, high-quality embryo, oocyte utilization rate, clinical outcome, live birth

## Abstract

**Objective:**

To explore the clinical application value of half-ICSI treatment for infertility in assisted reproductive technology.

**Method:**

A retrospective analysis of 1130 half-ICSI treatments was conducted at the Affiliated Reproductive Hospital of Shandong University from January 2011 to December 2015. Patients with low fertilization rates in previous cycles, primary infertility for >5 years with unexplained reason, or secondary infertility for >5 years without fallopian tube factor were involved in this study. The 2PN rate, high-quality embryo rate, oocyte utilization rate, and clinical outcomes were compared between IVF insemination group (IVF group) and ICSI insemination group (ICSI group). The clinical outcome of half-ICSI insemination treatment, grouped according primary and secondary infertility, was also analyzed.

**Results:**

Compared with IVF, ICSI resulted in a significantly higher 2PN rate (74.8% vs. 62.9%), high-quality embryo rate (54.6% vs. 51.7%), and oocyte utilization rate (35.9% vs. 32.8%; P<0.05). Among the 884 fresh-embryo transfer cycles, there were no notable differences in clinical pregnancy rate, live birth rate, or neonatal abnormality rate between the IVF and ICSI groups. Among the 792 primary infertility cycles, ICSI resulted in a significantly higher 2PN rate, high-quality embryo rate, and oocyte utilization rate compared with IVF (75.3% vs. 62.4%, 54.3% vs. 50.8%, 36.4% vs. 32.6%, P<0.05). For the 338 secondary infertility cycles, ICSI resulted in a significantly higher 2PN rate (73.6% vs. 63.9%, P<0.05) compared with IVF, but there were no notable differences in other laboratory results. Moreover, the biochemical pregnancy rate of the ICSI group was significantly lower than for IVF in secondary infertility cycles (49.3% vs. 65.6%; P<0.05). A total of 89 cycles (7.9%) with complete IVF fertilization failure showed a low second polar body (2PB) rate (33.6%) after a 5-h short-time fertilization period, including 34 cycles (3.0%) with no 2PB oocytes observed in the IVF group.

**Conclusion:**

ICSI insemination improved laboratory results compared with IVF insemination, however, fresh-embryo transfer of ICSI originated embryos did not improve clinical pregnancy and live birth rates. Rescue ICSI has been successfully applied in clinical IVF insemination to avoid fertilization failure. Therefore, as an extra intervention, it is suggested that ICSI be used judiciously.

## Introduction

Oocyte fertilization is a critical step in assisted reproductive technology (ART). A low fertilization rate or complete fertilization failure may occur in some infertility treatment cycles, and the subsequent repeated assisted pregnancy therapy may result in psychological and economic pressure on patients. The incidence of complete fertilization failure ranges from 10% to 20% (1). In 2003, Jaroudi et al. (2) first expressed that intracytoplasmic sperm injection (ICSI) and conventional *in vitro* fertilization (IVF) are complementary techniques in the management of unexplained infertility. Nevertheless, despite the high fertilization rate (3), it is still not recommended that ICSI be a blanket fertilization method in ART, as ICSI treatment is more invasive and costly than IVF.

Recently, increasing evidence has shown the positive role of half-ICSI in ART. Half-ICSI results in more high-quality embryos for transfer and improves the rate of pregnancy for patients with a high risk of fertilization failure (4). In 2010, Guo et al. (5) reported that half-ICS treatment may be useful for patients with unexplained infertility and primary infertility, but not for patients with oligo-asthenozoospermia, teratozoospermia, or secondary infertility. However, controversy exists between some studies. Sauerbrun-Cutler et al. (6) report that in a split sibling oocyte cohort, although ICSI had a higher fertilization rate and more high-quality day-2 embryos, it had a lower blastulation rate.

The purpose of this study was to further evaluate the effect of half-ICSI treatment in ART. We conducted a retrospective analysis of 1130 half-ICSI insemination treatments at our center from January 2011 to December 2015. We evaluated the effects of different insemination methods on the clinical outcomes of these patients, in order to provide a reference for more focused clinical treatment in the future.

## Materials and Methods

### Patients

A total of 1130 half-ICSI patients were enrolled in this study from January 2011 to December 2015. Half-ICSI treatment was given to patients with the following infertility backgrounds: patients with a fertilization rate between 30% and 50% in previous IVF cycles; patients with primary unexplained infertility for >5 years, or secondary infertility for >5 years without fallopian tube problems. All the patients involved in half-ICSI treatments had at least eight oocytes retrieved and the semen profile was normal.

### Ovarian Stimulation

All patients underwent controlled ovarian hyperstimulation(COH). Ovarian stimulation protocols included controlled ovarian hyperstimulation after gonadotropin-releasing hormone (GnRH) agonist down-regulation or an antagonist protocol. Recombinant follicle-stimulating hormone (rFSH, PUREGON; MSD Organon, Oss, Netherlands) was started on day 1–3 of the menstrual cycle. The dose adjustment of gonadotropin, monitoring of the ovarian response, and the timing for triggering the final oocyte maturation during ovarian stimulation was performed under the discretion of the supervising clinician. Oocyte retrieval was performed 34–36 h after the administration of human chorionic gonadotropin (hCG) at a dose of 4000–10 000 IU.

### Oocyte Insemination, Embryo Culture, and Embryo Transfer

Oocytes were inseminated approximately 3–6 h after follicular aspiration using a conventional insemination method and ICSI. The oocytes were divided into two groups equally and randomly. One group underwent IVF insemination and the other group underwent ICSI insemination. If the total number of oocytes is odd, one more oocyte was divided into the ICSI group. Short-time insemination in the IVF group was used, but no rescue ICSI was performed on the oocytes. Sequential culture media from Vitrolife (G-IVF, G1 and G2; Scandinavian IVF Science, Goteborg, Sweden) were used in all steps. Embryos were cultured separately in pre-equilibrated culture media overlaid with mineral oil. The culture dishes were housed in 37 °C tri-gas tabletop incubators (K-system, Denmark) containing 5% O_2_ and 6% CO_2_, balanced with N_2_. Two or three high-quality embryos were selected for fresh transfer on day 3. For patients who could only accept a single embryo transfer, a single blastocyst was selected and transferred on day 5. High-quality embryos in the IVF group were selected for transfer as a priority. High-quality embryos in the ICSI group were selected for transfer if there was only one or no high-quality embryo in the IVF group. Supernumerary embryos were cultured for blastocyst cryopreservation. Morphologic criteria were used for day-3 embryo scoring based on the amount of anucleate fragments expelled during early cleavage, and on developmental speed (7). Embryos transferred and cryopreserved by vitrification on day 5 were assessed to be above grade 4BC according to Garden and Lane criteria (8). Embryo transfer was performed using a Wallace catheter under ultrasound guidance.

### Outcome Measures

In both the ICSI insemination group (ICSI group) and the IVF insemination group (IVF group), the 2PN rate of matured oocytes, the high-quality day-3 embryo rate, and the utilized oocyte rate, including embryos transferred and embryos vitrified, were calculated.

When embryos originated from different insemination groups in fresh-embryo transfer cycles, the clinical results were calculated separately. All the fresh-embryo transfer cycles were divided into three groups: the IVF group, where IVF insemination embryos were transferred; the ICSI group, where ICSI insemination embryos were transferred; and the IVF plus ICSI group, where IVF embryos and ICSI embryos were both transferred at the same time. A serum hCG level>10 IU/L at 14 days after embryo transfer was diagnosed as a biochemical pregnancy and cardiac activity 7 weeks after embryo transfer was defined as a clinical pregnancy. Live birth was defined as the delivery of a live-born infant at≥28 weeks of gestation. Preterm birth rate and neonatal abnormalities were also calculated.

### Statistical Analysis

All analyses were performed using SPSS Statistics (version 22.0). Statistical analyses were conducted using the t-test and chi-square test. P-value was bilateral and P<0.05 was considered statistically significant. Statistical analysis was performed using a χ2 test. A P-value<0.05 was considered statistically significant.

## Results

### Description of the Study Patients

Patient characteristics are listed in **Table 1**. A total of 1130 patients were involved in this study. Their average age was 32.4 ± 4.1. Of these, 792 cases were primary infertility and 382 cases were secondary infertility. Finally, 884 cycles from these patients underwent fresh embryo transplantation. Cryopreservation was performed in 202 cycles, and 45 cycles were completely abandoned with no embryo transferred or frozen. The ovarian stimulation protocols used in the study included super long-term, long-term, short-term, antagonist, microstimulation protocol, and other protocols.

**Table 1:** Characteristics of the 1130 half-ICSI patients.

Characteristic	No. cycles	Value
Age (y) (mean ± STD)		32.43 ± 4.05
Infertility factors		
Primary	792 (70.1%)	
Secondary	338 (30.0%)	
Regimen of ovarian hyperstimulation		
super long-term protocol	12 (1.1%)	
long-term protocol	954 (84.4%)	
short-term protocol	104 (9.2%)	
Antagonist protocol	41 (3.6%)	
other protocol	16 (1.4%)	
microstimulation protocol	3 (0.3%)	
Result of treatment		
Fresh embryo-transfer cycles	884 (78.2%)	
Cryopreserved cycles	202 (17.9%)	
Complete abandoned cycles	45 (4.0%)	

### Embryo Development Analysis of the Half-ICSI Treatment Cycles

The 2PN rate, high-quality embryo rate, and utilized oocyte rate of ICSI embryos were significantly higher than for IVF embryos (74.8%vs. 62.9%, 54.6%vs. 51.7%, 35.9%vs. 32.8%, P<0.05; **Table 2**). Additionally, for the 792 primary infertility patients, the 2PN rate, high-quality embryo rate, and utilized oocyte rate of ICSI embryos were significantly higher than for IVF embryos (75.3%vs. 62.4%, 54.3%vs. 50.8%, 36.4%vs32.6%, P<0.05). For the 338 secondary infertility patients, the 2PN rate of ICSI insemination embryos was significantly higher than for IVF insemination embryos (73.6%vs. 63.9%, P<0.05), but the high-quality embryo rate and utilized oocyte rate did not differ between IVF embryos and ICSI embryos (**Table 3**).

**Table 2:** Embryo outcome of the total 1130 half-ICSI patients.

Characteristic	IVF	ICSI
Matured oocytes: no.	6728	7014
2PN: no. (%)	4230 (62.9)	5245 (74.8)^*^
High-quality embryo: no. (%)	2185 (51.7)	2862 (54.6)^*^
Transferred embryo: no.	978	782
Vitrified embryo: no.	1227	1737
Utilized oocytes: no. (%)	2205 (32.8)	2519 (35.9)^*^

Values are presented as number (%).

^*^P <0.05 ICSI compared to IVF groups.

**Table 3:** Embryo outcome of the 792 primary infertility and 338 secondary infertility patients.

	Primary infertility		Secondary infertility	
Characteristic	IVF	ICSI	IVF	ICSI
Matured oocytes: no.	4752	4947	1976	2067
2PN: no. (%)	2967 (62.4)	3724 (75.3)^*^	1264 (63.9)	1521 (73.6)^*^
High-quality embryo: no. (%)	1507 (50.8)	2021 (54.3)^*^	678 (53.7)	841 (55.3)
Transferred embryo: no.	650	547	328	235
Vitrified embryo: no.	897	1256	330	481
Utilized oocytes: no. (%)	1547 (32.6)	1803 (36.4)^*^	658 (33.3)	716 (34.6)

Values are presented as number (%).

^*^P <0.05 ICSI compared to IVF groups.

### Clinical Outcome Analysis of the Half-ICSI Treatment Cycles

A total of 884 patients underwent fresh embryo transfer. Biochemical pregnancy rates of the ICSI group and IVF plus ICSI group were both lower than for the IVF group (53.6%vs. 64.2%, 55.5%vs. 64.2%, P<0.05; **Table 4**). However, the clinical pregnancy rate, live birth rate, preterm birth rate, and the neonatal abnormality rate did not differ among the three groups. For primary infertility patients, all clinical indexes showed no clear difference. For secondary infertility patients, the biochemical pregnancy rate of the ICSI group and IVF plus ICSI group were both lower than for the IVF group (49.3%vs. 65.5%, 47.6%vs. 65.5%, P<0.05), and the clinical pregnancy rate, the live birth rate, and the preterm birth rate did not differ among the three groups (**Table 5**). There was no notable difference in neonatal abnormalities among the different transfer groups. The clinical outcome of patients with fresh embryo transplantation ≤ 35 years old was also analyzed (**Supplementary Tables 1**, **2**). The result is in accordance with the total fresh embryo transfer infertility patients.

**Table 4:** Clinical outcome of half-ICSI patients with fresh embryo-transfer cycles.

Characteristic	IVF	ICSI	IVF+ICSI
Fresh embryo transfer cycles	363	274	247
No. fresh embryo transferred per cycle (mean ± STD)	2.0 ± 0.3	1.9 ± 0.5	2.2 ± 0.4
Age (y) (mean ± STD)	32.5 ± 3.8	32.4 ± 4.1	33.0 ± 4.5
Live birth: no. (%)	164 (45.2)	114 (41.6)	100 (40.5)
Singleton live birth per woman	115 (31.7%)	86 (31.4%)	71 (28.7%)
Twin live birth per woman	49 (13.5%)	28 (10.2%)	29 (11.8%)
Biochemical pregnancy: no. (%)	233 (64.2)	147 (53.6)^a^	137 (55.5)^b^
Clinical pregnancy: no. (%)	200 (55.1)	132 (48.2)	120 (48.6)
Preterm birth [no./total no. (%)]	11/164 (6.7)	9/114 (7.9)	6/100 (6.0)
Neonatal abnormalities [no./total no. (%)]	5/213 (2.3)	4/142 (2.8)	2/129 (1.6)

Values are presented as number (%).

^a^P<0.05 ICSI compared to IVF groups.

^b^P<0.05 IVF+ICSI compared to IVF groups.

**Table 5:** Clinical outcome of primary infertility and secondary infertility half-ICSI patients with fresh embryo-transfer cycles.

	Primary infertility (608 cycles)			Secondary infertility (276 cycles)		
Characteristic	IVF	ICSI	IVF+ICSI	IVF	ICSI	IVF+ICSI
Fresh embryo transfer cycle: no. (%)	244 (40.1)	201 (33.1)	163 (26.8)	119 (43.1)	73 (26.4)	84 (30.4)
No. fresh embryo transferred per cycle (mean ± STD)	2.0 ± 0.3	1.9 ± 0.4	2.1 ± 0.3	2.0 ± 0.2	1.9 ± 0.5	2.2 ± 0.4
Age (y) (mean ± STD)	32.0 ± 3.7	31.9 ± 4.0	32.7 ± 4.5	33.6 ± 3.8	33.7 ± 4.0	33.8 ± 4.5
Live birth: no. (%)	107 (43.9)	86 (42.8)	66 (40.5)	57 (47.9)	28 (38.4)	34 (40.5)
Singleton live birth per woman	75 (30.7%)	65 (32.3%)	48 (29.4%)	40 (33.6%)	21 (28.8%)	23 (27.4%)
Twin live birth per woman	32 (13.2%)	21 (10.5%)	18 (11.1%)	17 (14.3%)	7 (9.6%)	11 (13.1%)
Biochemical pregnancy: no. (%)	155 (63.5)	111 (55.2)	97 (59.5)	78 (65.5)	36 (49.3)^a^	40 (47.6)^b^
Clinical pregnancy: no. (%)	135 (55.3)	100 (49.8)	81 (49.7)	65 (54.6)	32 (43.8)	39 (46.5)
Preterm birth: [no./total no. (%)]	6/107 (5.6)	5/86 (5.8)	3/66 (4.5)	5/57 (8.8)	4/28 (14.3)	3/34 (8.8)
Neonatal abnormalities: [no./total no. (%)]	5/139 (3.6)	3/107 (2.8)	2/84 (2.4)	0/74 (0.0)	1/35 (2.9)	0/45 (0.0)

Values are presented as number (%).

^a^P<0.05 ICSI compared to IVF groups.

^b^P<0.05 IVF+ICSI compared to IVF groups.

### Embryo and Clinical Outcome of the 89 Complete IVF Fertilization Failure Cycles

A total of 89 cycles (7.9%) with complete IVF fertilization failure showed a low second polar body expulsion (2PB) rate (33.6%) after a 5-h short-time fertilization period, which included 34 cycles (3.0%) with no 2PB oocytes observed in the IVF group. The 2PN rate, high-quality embryo rate and utilized oocyte rate of ICSI embryos were 71.1%, 62.9%, and 45.0%, respectively. The live birth rate, biochemical pregnancy rate, clinical pregnancy rate, preterm birth rate, nd the neonatal abnormality rate were 47.1%, 60%,55.7%, 3.0%, and 4.9%, respectively (**Table 6**).

**Table 6:** Embryo and clinical outcome of 89 cycles of complete IVF infertility failure.

Characteristic	IVF	ICSI
No. of matured oocytes	417	478
2PN: no. (%)	0	340 (71.1)
High-quality embryo: no. (%)	0	214 (62.9)
No. of embryos transferred	0	124
No. of vitrified embryos	0	91
Utilized oocytes: no. (%)	0	215 (45.0)
Fresh embryo transfer cycle	0	70
Live birth: no. (%)	–	33 (47.1)
Singleton live birth per woman	–	25 (35.7%)
Twin live birth per woman	–	8 (11.4%)
Biochemical pregnancy: no. (%)	–	42 (60)
Clinical pregnancy: no. (%)	–	39 (55.7)
Preterm birth: [no./total no. (%)]	–	1/33 (3.0)
Neonatal abnormalities: [no./total no. (%)]	–	2/41 (4.9)
Second polar body expulsion: no. (%)	140(33.6)	

## Discussion

Oocyte fertilization is a complex process affected by a series of factors, including state of oocyte maturation, sperm maturation, and vitality or fusion of genetic material. Any abnormality in these steps leads to fertilization failure. For oligozoospermia and asthenozoospermia patients, ICSI greatly improves oocyte fertilization rate. In recent years, several studies have found that ICSI fertilization can also be effective in improving the fertilization rate of patients with unexplained infertility (3). Based on existing research, we adopted the half-ICSI treatment for some patients. The selected patients exhibited: primary or secondary fertilization failure for >5 years, or had a low IVF fertilization rate in past cycles. Our results showed that for primary infertility patients, ICSI resulted in a significantly higher 2PN rate, high-quality embryo rate, and oocyte utilization rate compared with the IVF group. However, biochemical pregnancy rate, clinical pregnancy rate, and live birth rate did not differ among the IVF group, ICSI group, and IVF plus ICSI group. The application of ICSI improved embryo quality but did not ultimately increase clinical pregnancy rate. We speculated that although ICSI guarantees embryo fertilization and early development, it does not improve the later developmental potential of embryos after transfer into the uterus.

For secondary infertility patients, ICSI only resulted in a significantly higher 2PN rate compared with IVF. The high-quality embryo rate and the utilized oocyte rate did not differ between IVF and ICSI groups. Moreover, ICSI embryos had a lower biochemical pregnancy rate. Therefore, according to our results, ICSI is not necessary for secondary infertility patients. Furthermore, the ICSI procedure may decrease embryo implantation capacity.

ICSI was first used for male-factor infertility, but in recent years, it has also been used in non-male-factor infertility cycles (9). The application of ICSI reduces the risk of complete fertilization failure, but does not increase the cumulative live birth rate in non-male factor infertility (10). Recently, accumulating information has shown the risk of ICSI on offspring, including congenital malformations, chromosomal abnormalities, and epigenetic syndromes (11–13). Cai et al. (14) reported that a sex ratio imbalance following blastocyst transfer is also associated with ICSI but not with IVF. A previous study also reported that for male-factor infertility, ICSI affects the fertility of male offspring, decreasing semen quality and quantity in young adults conceived by ICSI (15). Considering these risk, ICSI should be used with caution.

Short-time insemination and immediate rescue ICSI have been widely used in recent years, which has decreased complete fertilization failure. ICSI can be performed on oocytes that do not discharge a second polar body within 4–6 hours post-insemination (16). In our study, a total of 89 cycles with complete IVF fertilization failure showed a low second polar body (2PB) expulsion rate (33.6%) after a 5-h short-time fertilization period, which included 34 cycles with no 2PB oocytes in the IVF group. For this group of patients, immediate rescue ICSI after short-time insemination could avoid complete IVF fertilization failure.

This retrospective study had a large sample size and was conducted at one IVF laboratory. The laboratory results were valuable in comparing the effect of ICSI and IVF treatment for sibling oocytes. However, based on the benefit to patients, the embryo transfer selection order decreased the clinical significance of this study. A further study on cumulative pregnancy rate in the future may provide more conclusive answers. A second limitation was the absence of exclusively IVF or exclusively ICSI matched groups based on the same infertility factors. Hence, further studies, including prospective, randomized, controlled trials, are required to evaluate the clinical significance of half-ICSI.

In conclusion, this study demonstrated that half-ICSI insemination may be successful for primary infertility patients; however, for secondary infertility patients, ICSI is not necessary and may be an excessive intervention. For patients with a lower fertilization failure rate in conventional IVF, the use of short-time insemination and rescue ICSI would be key. With concern for the safety of ART, we suggest that half-ICSI is not necessary for patients with normal semen and that ICSI should be used with more caution.

## Data Availability Statement

The raw data supporting the conclusions of this article will be made available by the authors, without undue reservation.

## Ethics Statement

The studies involving human participants were reviewed and approved by the Ethics Committee of Center for Reproductive Medicine, Cheeloo College of Medicine, Shandong University.

## Author Contributions

ML put forward the study question and designed the research. NP analyzed the data. NP and SM drafted the manuscript and ML revised it critically. All authors were involved in the acquisition of data and have approved the version to be published.

## Funding

The National Natural Science Foundation of China (81401266), The National Natural Science Foundation of China (82171842) and the Natural Science Foundation of Shandong Province (ZR2013HQ053). The funding sources had no role in the study design, collection, analysis, or interpretation of the data, writing the manuscript, or the decision to submit the paper for publication.

## Conflict of Interest

The authors declare that the research was conducted in the absence of any commercial or financial relationships that could be construed as a potential conflict of interest.

## Publisher’s Note

All claims expressed in this article are solely those of the authors and do not necessarily represent those of their affiliated organizations, or those of the publisher, the editors and the reviewers. Any product that may be evaluated in this article, or claim that may be made by its manufacturer, is not guaranteed or endorsed by the publisher.
