# Supplementary material for: Intracytoplasmic Sperm Injection May Not Improve Clinical Outcomes Despite Its Positive Effect on Embryo Results: A Retrospective Analysis of 1130 Half-ICSI Treatments
Source: Front Endocrinol (Lausanne). 2022 Jun 15;13:877471. doi: 10.3389/fendo.2022.877471 (PMC9240197; doi:10.3389/fendo.2022.877471)
Supplement: Supplementary file 1 [file DataSheet_1.pdf]

Supplement table 1. Clinical outcome of half-ICSI patients ≤ 35 years old with fresh embryo-transfer cycles

Characteristic	IVF	ICSI	IVF+ICSI
Fresh embryo transfer cycles	281	207	173
No. fresh embryo transferred per cycle (mean±STD)	2.0 ± 0.3	1.9 ± 0.4	2.1 ±0.3
Age (y) (mean±STD)	31.0 ± 2.7	30.6 ± 2.8	30.8 ± 3.0
Live birth: no. (%)	131 (46.6)	88 (42.5)	79 (45.7)
Singleton live birth per woman	89 (31.7)	62 (30.0)	56 (32.4)
Twin live birth per woman	42 (14.9)	26 (12.5)	23 (13.3)
Biochemical pregnancy: no. (%)	187 (66.5)	113 (54.6) [a]	99 (57.2) [b]
Clinical pregnancy: no. (%)	157 (55.9)	102 (49.3)	89 (51.4)
Preterm birth [no./total no. (%)]	6/131 (4.6)	6/88 (6.8)	6/79 (7.6)
Neonatal abnormalities [no./total no. (%)]	4/173 (2.3)	4/114 (3.5)	2/102 (2.0)

Note: Values are presented as number (%).
[a]P<0.05 ICSI compared to IVF groups.
[b]P<0.05 IVF+ICSI compared to IVF groups.

Supplement table 2. Clinical outcome of primary infertility and secondary infertility half-ICSI patients ≤ 35 years old with fresh embryo-transfer cycles

	Primary infertility (481)			Secondary infertility (180)		
Characteristic	IVF	ICSI	IVF+ICSI	IVF	ICSI	IVF+ICSI
Fresh embryo transfer cycle: no. (%)	201 (41.8)	161 (33.5)	119 (24.7)	80 (44.4)	46 (25.6)	54 (30.0)
No. fresh embryo transferred per cycle (mean ± STD)	2.0 ± 0.3	1.8 ± 0.4	2.1 ± 0.3	2.0 ± 0.3	1.9 ± 0.5	2.1 ±0.4
Age (y) (mean ± STD)	30.8 ± 2.8	30.4 ± 2.8	30.6 ± 3.1	31.5 ± 2.7	31.2 ± 2.7	31.1 ± 2.8
Live birth: no. (%)	91 (45.3)	69 (42.9)	56 (47.1)	40 (50)	19 (41.3)	22 (40.7)
Singleton live birth per woman	62 (30.8)	50 (31.1)	39 (32.8)	27 (33.8)	12 (26.1)	16 (29.6)
Twin live birth per woman	29 (14.5)	19 (11.8)	17 (14.3)	13 (16.2)	7 (15.2)	6 (11.1)
Biochemical pregnancy: no. (%)	131 (65.2)	89 (55.3)	74 (62.2)	56 (70.0)	24 (52.2) [a]	25 (46.3) [b]
Clinical pregnancy: no. (%)	112 (55.7)	81 (50.3)	65 (54.6)	45 (56.3)	21 (45.7)	24 (44.4)

Preterm birth: [no./total no. (%)]	3/91 (3.3)	3/69 (4.3)	3/56 (5.4)	3/40 (7.5)	3/19 (15.8)	3/22 (13.6)
Neonatal abnormalities: [no./total no. (%)]	4/120 (3.3)	3/88 (3.4)	2/73 (2.7)	0/53 (0)	1/26 (3.8)	0/28 (0)

Note: Values are presented as number (%).

[a]P<0.05 ICSI compared to IVF groups.

[b]P<0.05 IVF+ICSI compared to IVF groups.
